# *Camelina sativa*: An Emerging Feedstuff for Laying Hens to Improve the Nutritional Quality of Eggs and Meat

**DOI:** 10.3390/ani15152173

**Published:** 2025-07-23

**Authors:** Yazavinder Singh, Antonella Dalle Zotte, Bianca Palumbo, Marco Cullere

**Affiliations:** Department of Animal Medicine, Production and Health—MAPS, University of Padova, Viale dell’Università 16, 35020 Legnaro, PD, Italy; yazavinder.singh@unipd.it (Y.S.); biancafederica.palumbo@phd.unipd.it (B.P.); marco.cullere@unipd.it (M.C.)

**Keywords:** *Camelina sativa*, feed, hen, poultry, egg, quality, omega-3, sustainability

## Abstract

**Simple Summary:**

The present research aimed at formulating a diet for free-range laying hens incorporating an emerging sustainable feedstuff, *Camelina sativa*, and testing its effectiveness in enhancing the omega-3 content of eggs and to possibly valorize the meat quality of hens at the end of the laying cycle. After 28 days of experiment, *Camelina sativa* allowed for the obtainment of eggs with the health claim “rich in omega-3 fatty acids” and with an overall quality and shelf-life comparable to those of hens fed with a conventional diet. Also, the meat of *Camelina sativa*-fed hens at the end of the laying season had an improved omega-3 fatty acids content which was, however, not sufficient to reach any nutritional claim. Overall, the results of the present research highlighted that *Camelina sativa* offers interesting perspectives in feed formulations for free-range laying hens.

**Abstract:**

*Camelina sativa* (CS) is an emerging sustainable oilseed crop with interesting feed application potentialities. The research assessed the potentiality of *Camelina sativa* (CS) in the diet for free-range laying hens, aiming at reaching a nutritional claim. To this purpose, two feeding groups of hens (*n* = 100 Livorno hens, *n* = 25 hens/pen) were farmed outdoor and received either a Control diet, which was a commercial diet for laying hens, or a CS diet. The latter was formulated to include the 5% CS cake and 1% CS oil, replacing conventional feedstuffs. Diets were isoprotein and isoenergy and were available *ad libitum* throughout the laying period (February–September). At day 1, *n* = 12 eggs/diet were sampled to assess the initial proximate composition and FA profile. Every 7 days the sampling was repeated to analyze the eggs’ FA profile, up to day 35. At the end of the laying season, *n* = 6 hens/dietary treatment were slaughtered and subjected to meat quality evaluations. Results highlighted that a 28-day feeding was the period required to obtain 227 mg of C18:3 *n*-3 and 81 mg of C20:5 *n*-3 + C22:6 *n*-3/100 g egg, whereas a further 7 days of feeding trial were ineffective in further enhancing the omega-3 FA content of eggs. CS eggs were comparable to Control ones for overall physical traits, proximate composition, and shelf-life. In addition, at the end of the laying period, the meat of CS hens was also found to be healthier than that of Control ones, thanks to a higher omega-3 FA proportion (*p* < 0.01), which was, however, not sufficient to reach any nutritional claim. Instead, the proximate composition of CS meat was overall comparable to Control hens. In conclusion, the present research demonstrated that feeding *Camelina sativa* meal and oil to laying hens is feasible and allows to reach the nutritional claim in eggs “rich in omega-3 FA” after a feeding period of 28 days, without any negative effects on other eggs’ quality trials. *Camelina sativa* can thus be defined as a promising sustainable feedstuff for the poultry sector for diversification purposes and to enhance the nutritional quality of eggs.

## 1. Introduction

The egg sector has been steadily growing worldwide in the last decades, from 26.2 to 86.7 million metric tons in the period 1980–2020, having a compound growth rate per year second only to that of chicken meat. In this scenario, Europe accounts for the 49.1% of the global production [1]. This market development can be explained by the rising demand for animal protein and the intrinsic nutritional properties and health benefits of eggs associated with the limited ethical–religious constraints towards their consumption [2,3]. In the European Union, official data indicate that most eggs come from hens farmed in enriched cages (44.9%), another 35.6% come from barn–aviary systems, and free-range and organic housing account for a 12.8% and 6.6%, respectively, with a great variability among EU countries [4].

Even if free-range still accounts for a relatively low percentage of EU housing systems, recent studies indicate that they are more and more perceived by consumers as the market choice to achieve a more sustainable egg supply chain. In addition, consumers (especially <40 years old ones) feel that free-range systems can provide better welfare conditions and overall quality of life to laying hens [5,6]. Together with the hens’ farming system, also the nutritional properties of eggs are another factor affecting the willingness to buy of a certain niche of consumers, especially when supported by labelling and appropriate communication strategies, contributing to create a solid market segment [7]. In this context, exploiting unconventional and sustainable feedstuffs to feed hens, besides achieving a better sustainability of the production, represents another driver to retain the above-mentioned consumers’ niche.

*Camelina sativa* (L) Crantz (CS) is an emerging oilseed crop of the *Brassicaceae* family, which has gained interest as feedstuff thanks to a series of positive agronomic and nutritional characteristics. These include a wide environmental adaptability, the resistance to draught, tolerance to pest and diseases and, thus, limited requirement of pesticides. In addition, the existence of winter and spring types allow for its inclusion into crop rotations. Also, CS has a high protein and oil content, the latter characterized by a healthy fatty acids profile being rich in omega-3 fatty acids [8].

Existing research applications of CS into poultry species, considering both meat [9,10,11] and egg [11,12] productions, were recently revised. Results display a wide variability: while the inclusion of CS oil as a replacement of conventional sources (mainly soybean and sunflower) does not seem to provide any disadvantage, as it enhances the omega-3 content of meat/eggs, the use of CS seeds or cake is more critical. In fact, CS naturally contains glucosinolates, phytic acid, sinapine, trypsin inhibitors, and condensed tannins, which are well known antinutritional factors. Among them, glucosinolates play a major role in worsening nutrient absorption-utilization. Factors like CS genetics, agronomic conditions, and seed treatments can affect the amounts of antinutritional factors, but it has generally been observed that 10% seeds/cakes represent a sort of upper threshold [9,10,11,12]. Of course, inclusion levels >10% guarantee a relevant enhancement of product healthiness, but, in most cases, at the expense of productive outcomes.

Focusing on the eggs produced by CS-fed hens, research published up to now was based on experimental designs with the following structure: testing one or more dietary CS inclusion levels, setting an experimental duration, analyzing the productive outcomes, and, at the end of the experiment, assessing the nutritional quality of the eggs [13,14,15,16,17]. Diversely, the impact of a CS diet was never assessed in the perspective to establish the minimum time required to achieve a product with an objectively established health benefit, namely “source or rich in omega-3 fatty acids”, in accordance with the Regulation (EC) No. 1924/2006 of the European Parliament and Council of 20 December 2006 on nutrition and health claims made on foods. In addition, it is still unclear if, considering a complete laying cycle, the meat of hens at the end of the laying season can also benefit from the dietary presence of CS, also in the perspective to comply with the above-mentioned EU Regulation on nutritional claims. The latter is another relevant aspect since the meat of spent hens has a low market value, and it is generally valorized through incorporation in processed ready-to-cook/eat poultry meat products such as sausages, spreads, etc. [18,19]. However, in this case, the farmer cannot economically benefit from these commercial applications and spent laying hens are mainly considered a waste material [20].

Therefore, considering the existing knowledge on the topic, the present research aimed at testing a specific inclusion level of *Camelina sativa* (CS) cake and oil in the diet of hens farmed in a free-range system to assess if it is possible to obtain eggs with a nutritional claim (source or rich in omega-3 fatty acids) without compromising other qualitative attributes. Another target is to establish, for the inclusion levels of tested CS products, the time required to achieve the claim. A third objective is to assess if, at the end of the laying season, the meat of hens could benefit from a potential market valorization exploiting a nutritional claim, which could generate an additional income for the farmer.

## 2. Materials and Methods

### 2.1. Experimental Design

The present research was conducted at a commercial farm located in Ceregnano (RO, Italy) after approval by the veterinary authority and according to the Article 2, DL 4 March 2014, No. 26, of the Official Journal of the Italian Republic (http://www.gazzettaufficiale.it/eli/id/2014/03/14/14G00036/sg—accessed on 25 June 2025), which implements the EU Directive 2010/63/EU on the protection of animals used for scientific purposes.

In February, a total of *n* = 100 Livorno hens at the beginning of the laying cycle (16 weeks of age) and present at the commercial farm were randomly housed in a free-range condition: they were in 4 outdoor pens (*n* = 25 hens/pen; each pen measured 4 m × 10 m), with free access to an indoor area to lay eggs, rest or hide. They were fed with a commercial diet (ingredients: corn, wheat middlings, decorticated sunflower meal, gluten corn meal, roasted and decorticated soybean meal, calcium carbonate, whole alfalfa meal, mono-dicalcium phosphate, soybean oil, sodium chloride) normally used at the farm (Control, pellet form). After 2 days the experiment started: *n* = 50 hens kept on eating the Control feed, while the other *n* = 50 hens received an experimental feed where *Camelina sativa* cake and oil were incorporated at 5% and 1% levels, respectively, replacing conventional feedstuffs (CS diet, pellet form): CS replaced 2% corn, 1% soybean meal, 1% sunflower meal, and 2% soybean oil (100% oil substitution). The CS inclusion levels were chosen based on existing research outcomes, as detailed in the introduction section. The *Camelina sativa* was sown, harvested, and processed (mechanical separation of the oil and protein fractions) in the same commercial farm where the experiment was carried out. The cake had the following chemical composition (g/kg, as is basis): 921 dry matter (DM), 281 crude protein (CP), 216 ether extract (EE), 59.9 Ash. The two diets were isoprotein and isoenergy, and they were available ad libitum throughout the laying period (February–September), as it was for drinking water. The chemical composition and fatty acids profile (FA) of the two diets are presented in Table 1 and Table 2, respectively.

Each pen had access to a covered poultry house where hens could enter to lay eggs and to rest during the night. Throughout the study, hens followed the natural photoperiod, and the environment inside the poultry house was not controlled. The outdoor pen had no pasture, so the sole source of feed for the laying hens was the experimental diets.

### 2.2. Sampling Procedures

The day the experiment started, just before providing the experimental diets, a total of *n* = 12 eggs/dietary group (*n* = 6 eggs/pen) were collected and dedicated to the following analyses: proximate composition and FA profile. This was to obtain important initial information, i.e., to assess the nutritional characteristics of the eggs at the beginning of the experiment. Subsequently, the same number of eggs (*n* = 12 eggs/dietary group; *n* = 6 eggs/pen) was sampled weekly for the FA analysis. Egg samplings ended at day 35, when the FA results indicated that no further omega-3 FA deposition in the eggs was occurring compared to the previous week, and, thus, a plateau had been reached at 28 days. Therefore, at day 28, when the highest incorporation level of omega-3 FA was observed, eggs were also subjected to the proximate composition analysis. At each sampling day, freshly collected eggs were transported to the Laboratory LabCNX of the Department of Animal Medicine, Production and Health—MAPS of the University of Padova (Italy). At the end of the experimental period, a further *n* = 84 eggs/dietary group were sampled in consecutive days for physical determinations and shelf-life trial, as it will be detailed in the next section.

### 2.3. Egg Quality

Once at the MAPS Department, eggs were individually broken, freeze-dried (Edwards, Minifast, Paignton, UK: freezing at −40 °C overnight and subsequent vacuum sublimation at +40 °C for 48 h) and ground (Retsch Grindomix GM 200, Haan, Germany: 5000 g for 5 s) to a fine powder to ensure sample homogeneity. Freeze-dried samples were dedicated to the following analyses: proximate composition and FA profile. Differently, physical traits and shelf-life were assessed on fresh eggs.

Proximate composition was analyzed on eggs collected at days 1 and 28 following the procedures of the Association of Official Analytical Chemists [21]: method no. 934.01 for Dry Matter (DM), method no. 2001.11 for Crude Protein (CP), and method no. 942.05 for ash. The EE was determined after acid hydrolysis [22].

For the FA analysis, analyzed at days 1, 7, 14, 21, 28, and 35, fat was extracted exploiting the binary mixture of solvents hexane/Isopropanol 3:2 by Accelerated Solvent Extraction (M-ASE), as detailed in Dalle Zotte et al. [23]. The sole exception regarded the gas chromatograph used to perform the analysis, which was a Shimadzu 2010 plus (Kyoto, Japan). The results were expressed as a % of total detected fatty acid methyl esters (FAME). In addition, the quantitative determination (mg/100 g egg) of FA was calculated by exploiting the chromatographic peak area and in accordance with the internal standard (nonadecylic acid—C19:0) and the total lipid content of the sample.

Eggs’ physical traits were analyzed at the end of the trial (egg collection from day 28): after weighing the egg, a digital caliper (Juwel PLUS, Wissen, Germany; 0–150 mm—Juwel) was used to measure the equatorial diameter (mm) and egg height (mm). The above-mentioned measurements were used to calculate the egg surface area (cm^2^) and the shape index (%). Afterwards, the fracture toughness of eggs was measured by using a Texture Analyzer, model TA-HDi (SMS—Stable Micro Systems, London, UK), as detailed in Dalle Zotte et al. [24]. After breaking the eggs, the eggshell was dried and weighed (±0.1 g), and eggshell thickness (mm) was measured at equatorial level with the above-mentioned digital caliper. Albumen and yolk were weighed to calculate the relative incidences, as well as the yolk/albumen ratio. Thanks to the physical measurement described previously, it was possible to obtain the edible portion (%).

For the shelf-life assessment, eggs were stored for 28 days in commercial egg packages at room temperature, avoiding direct light. This was set to simulate retail display conditions. At the beginning and end of the shelf-life, the following analyses were performed: albumen pH, yolk color, and lipid oxidation. The pH of the albumen (±0.1) was determined in duplicate by using a FG2-Five GoTM (Mettler Toledo, Greifensee, Switzerland—calibrated at pH 4, 7, and 10), while yolk color was evaluated by the 16-scale color index dsm-firmenich YolkFan™ (DSM, Kaiseraugst, Switzerland). The egg yolk of each egg was then used to analyze the lipid peroxidation (malondialdehyde-MDA equivalents: mg MDA/kg egg yolk), which was assessed using a spectrophotometer (Hitachi U-2000; Hitachi, Mannheim, Germany) set at 532 nm. The instrument measured the absorbance of a 1,1,3,3-tetraethoxypropane calibration curve and of thiobarbituric acid-reactive substances—TBARs [25].

### 2.4. Hen’s Meat Quality

At the end of the laying season (September), *n* = 6 hens/dietary group (*n* = 3 hens/pen) were electrically slaughtered at a commercial abattoir following the standard procedure and then transported to the MAPS Department (+4 °C) for meat quality evaluations. After deboning, meat of the whole carcass was ground (Retsch Grindomix GM 200: 7000 g for 10 s), freeze-dried, and dedicated to the following analyses: proximate composition and FA profile. Proximate and FA analyses were performed following the same procedures described for eggs. The sole exception was the binary solvent mixture that was used to extract lipids (chloroform:methanol—1:2) for the subsequent FA profile determination and FA content calculations.

### 2.5. Statistical Analysis

Data concerning eggs’ physicochemical traits were processed with a one-way ANOVA with the experimental diet (Control vs. CS) as fixed effect following the General Linear Model (GLM) procedures of the SAS 9.1.3 statistical analysis software for Windows [26]. For the shelf-life trial, a two-way ANOVA tested the effects of the dietary group, day of storage, and their interaction. The experimental unit was the single egg. Least square means were obtained by post-hoc pairwise comparisons using the Bonferroni test, and the significance level was calculated at a 5% confidence threshold.

## 3. Results

At the beginning of the feeding experiment, eggs belonging to the two experimental groups showed a comparable proximate composition (Table 3): 15.5 g protein/100 g egg, 7.83 g lipids/100 g egg, and 0.86 g ash/100 g egg. A similar egg proximate composition in the two experimental groups was observed also at the end of the experiment, after 28 days: 15.0 g protein/100 g egg, 8.04 g lipids/100 g egg, and 0.86 g ash/100 g egg.

The FA content of eggs analyzed at the beginning of the feeding trial (Table 4) showed no differences in the two dietary treatments. Eggs had saturated (SFA), monounsaturated (MUFA), and polyunsaturated (PUFA) contents of 2510, 3310, and 1650 mg/100 g egg, respectively (average values of the two groups). The *n*-3 and the EPA + DHA FA contents were 119 and 46 mg/100 g egg, respectively (average values of the two groups).

**Figure 1:**
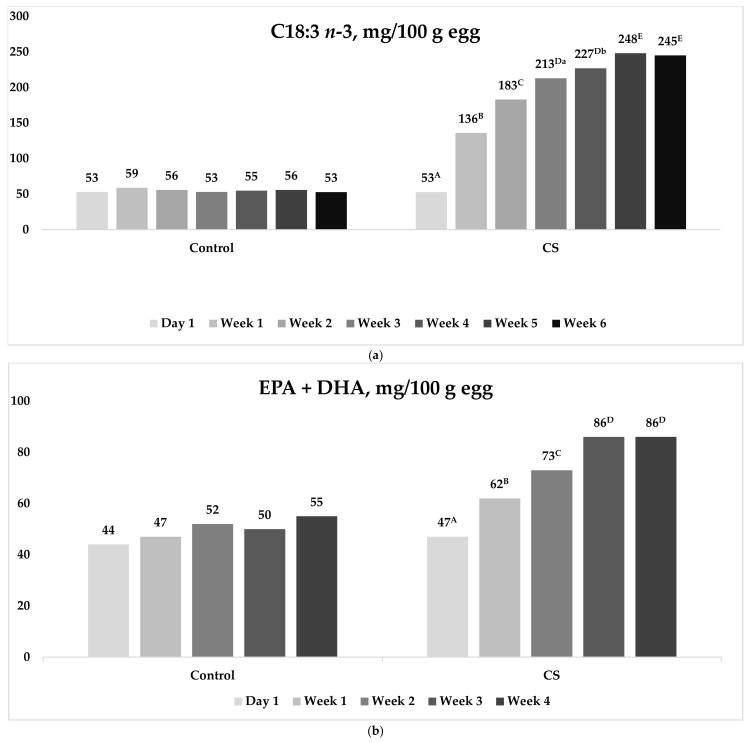
(**a**,**b**) Effect of the inclusion of *Camelina sativa* (CS, cake and oil) in laying hens’ diets on the C18:3 *n*-3 and C20:5 *n*-3 + C22:6 *n*-3 (EPA + DHA) FA contents (mg/100 g egg) from the beginning of the experiment, up to the incorporation plateau. ^A, B, C, D, E^ Different superscript letters differ for *p* < 0.01; ^a, b^ Different superscript letters differ for *p* < 0.05.

The dietary incorporation of *Camelina sativa* cake and oil into laying hens’ diet had a profound effect on eggs’ FA proportions along the experiment (Figure 1a,b), as well as on the content, analyzed after 28 days of feeding experiment (Table 5). Figure 1 shows the effect of the inclusion of *Camelina sativa* (CS, cake and oil) in laying hens’ diets on the C18:3 *n*-3 (Figure 1a) and C20:5 *n*-3 + C22:6 *n*-3 (EPA + DHA; Figure 1b) FA contents (mg/100 g egg) from the beginning of the experiment, up to the deposition plateau. In the case of α-linolenic acid, CS eggs highlighted a constant increase (from 53 to 248 mg/100 g egg in CS eggs; *p* < 0.01), and the deposition peak was reached after 5 weeks of dietary treatment. Also, the sum of EPA and DHA increased along with the experimental period (from 47 to 86 mg/100 g egg; *p* < 0.01), but in this case, the plateau was reached after 4 weeks. Thanks to the EPA + DHA sum, CS eggs reached the nutritional claim “rich in omega-3 FA”. Diversely, Control eggs displayed constant amounts of C18:3 *n*-3 (average: 55 mg/100 g egg) and EPA + DHA (average: 50 mg/100 g egg) throughout the experiment.

While the SFA remained similar in Control and CS eggs, a small change in the MUFA fraction was observed: CS eggs showed a higher content of C20:1 *n*-9 compared to Control ones (*p* < 0.001). The observed change of this FA proportion in the two groups was, however, not sufficient to determine a significant outcome in the overall MUFA amount of the two dietary treatments. The dietary inclusion of *Camelina sativa* cake and oil strongly affected that PUFA amounts of the eggs, especially of the *n*-3 fraction: C18:3 *n*-3 (54.9 vs. 227 mg/100 g egg in Control and CS groups, respectively; *p* < 0.001), C20:3 *n*-3 (0.00 vs. 8.86 mg/100 g egg in Control and CS groups, respectively; *p* < 0.001), and C22:6 *n*-3 (EPA: 54.8 vs. 85.5 mg/100 g egg in Control and CS groups, respectively; *p* < 0.001) were higher in CS than in Control eggs. Overall, the amount of *n*-3 in CS eggs was about three times that of the Control group (110 vs. 313 mg/100 g eggs in the Control and CS groups, respectively; *p* < 0.001). Also, the CS diet lowered the C18:2 *n*-6 content of eggs compared to the Control, which determined a lower overall *n*-6 amount in CS compared to Control eggs (*p* < 0.001).

Table 6 presents the effect of the studied dietary treatment on the physical characteristics of eggs at 28 days of the feeding trial. Overall, the dietary incorporation of *Camelina sativa* cake and oil into a hen’s diet did not affect eggs’ physical traits. The sole exceptions were shape index (*p* < 0.01) and fracture toughness (*p* < 0.01). In both traits, values were higher in CS eggs compared to Control ones (shape index: 75.3 vs. 73.9% in CS and Control eggs, respectively; fracture toughness: 45.2 vs. 39.6 N in CS and Control eggs, respectively). Conversely, yolk, albumen, and shell characteristics did not change in the experimental groups.

Eggs collected at the end of the feeding trial were subjected to a 28-day retail display, whose results are depicted in Table 7. The dietary treatment did not affect the storage stability of eggs, as albumen pH and yolk color showed similar outcomes in the Control and CS groups. The exception was the oxidative stability of lipids, as, after 28 days of storage, the TBARs value was higher in CS eggs compared to Control ones (0.78 vs. 0.83 mg MDA/kg egg for Control and CS groups, respectively; *p* < 0.001). Considering the day effect, eggs’ physicochemical characteristics varied from day 0 to day 28: albumen pH increased along with storage in both groups (*p* < 0.001), and a partial yolk discoloration was recorded (*p* < 0.001). Regarding the oxidative status of egg lipids, TBARs value increased with storage in the CS group (*p* < 0.001) but not in the Control one (*p* > 0.05).

At the end of the laying season, the meat quality of spent hens was evaluated for its nutritional characteristics: Table 8 shows the proximate composition, while Table 9 presents the FA content. Hens fed the CS diet had a meat poorer in protein (*p* < 0.01) compared to that of the Control, with other traits remaining unaffected (*p* > 0.05). Despite the limited lipids content of the meat, the FA amounts slightly differed in the two experimental groups: the CS diet increased the content of C18:3 *n*-3 (17.4 vs. 56.0 mg/100 g meat in Control and CS group, respectively; *p* < 0.05), which led to a higher overall *n*-3 FA amount, three times higher in the CS group than in the Control one (21.5 vs. 62.1 mg/100 g meat in Control and CS group, respectively; *p* < 0.05). In addition, the meat of CS hens had 1.13 mg/100 g meat of C20:1 *n*-9, while the same FA was absent in the Control group (*p* < 0.01).

## 4. Discussion

Results of the present research confirmed the potential of *Camelina sativa* for laying hens’ diet, which is coherent with the experimental outcomes obtained from different food-producing animal species [27]. The dietary inclusion of *Camelina sativa* cake (5%) and oil (1%) did not affect egg proximate composition and physical traits after a 28-day feeding experiment, and it agrees with the existing research on laying poultry species [12]. Relative to the observed different shape index and fracture toughness observed in CS eggs compared to the Control ones, the result seems apparently contradictory: in fact, CS eggs were more elongated than Control ones, which is generally associated with a lower resistance of the eggshell to fracture [28]. However, it was observed exactly the opposite since CS eggs displayed a higher fracture toughness than those of the Control group. This apparent incoherent result was attributable to two different factors: the shape index of chicken eggs can be classified as sharp (<72), normal (72–76), or round (>76), and, according to the present results, both Control and CS eggs fell within the normal range [29]. Furthermore, another aspect to be considered is the Ca content of the diets, which was 31,447 vs. 36,075 mg/kg in Control and CS diets, respectively. Ca is directly involved in eggshell formation, and past research on 23-week-old Hy-Line W-98 hens indicated that the optimum dietary level should be comprised within 4.34–4.62% [30]. Compared to such values, diets of the present research were both deficient in Ca (3.15% and 3.61% in Control and CS diets, respectively), probably contributing to explaining the result on eggshell fracture toughness since the CS diet had a higher Ca amount.

*Camelina sativa* is a rich source of α-linolenic acid (C18:3 *n*-3, ALA), whose proportion ranges between 28 and 50% of total FA [8]. In fact, the CS diet had a higher percentage of ALA compared to the Control one (12.3 vs. 3.37% of total FAME), which led to a consistently lower *n*-6/*n*-3 (3.40 vs. 13.4 in CS and Control diets, respectively). When exploiting dietary strategies to enhance the omega-3 content of eggs, there are several factors to be taken into account. Besides the genetics and the age of the hens [31], the omega-3 FA composition of the feedstuff, as well as its amount of omega-6 FA and the presence (and quantity) of antinutritional factors, play a pivotal role. In fact, it was observed that an excessive presence of flaxseed in the experimental diet caused a higher passage rate of the digesta compared to that of the control group. This, together with lowering the apparent metabolizable energy of the diet, caused a lower absorption of omega-3 FA [32].

In addition to the accumulation of ALA, which should increase along with the inclusion level of the ALA-rich feedstuff, hens are also capable of partly converting it into DHA in the liver. In fact, CS eggs reached the nutritional claim “rich in omega-3 FA” thanks to the EPA + DHA sum. However, the accumulation of this FA in the yolk in response to an increased dietary ingestion of ALA is not linear [33], which was confirmed also in the present study. In fact, ALA increased from 53 mg/100 g egg to 248 mg/100 g egg in 5 weeks, whereas EPA + DHA went from 47 mg/100 g egg to 86 mg/100 g egg in 4 weeks. The reason for this nonlinear conversion is primarily attributable to the presence of omega-6 FAs in the diet, because they are known to compete for the desaturase enzymes, which are fundamental for ALA conversion. In this regard, the dietary *n*-6/*n*-3 is a key indicator, and a lower ratio indicates a lower competition for the desaturase enzymes and, thus, a potentially higher ALA conversion efficiency. The delicate equilibrium between dietary omega-6 and omega-3 amounts and consequent competition for enzymes explains the different egg amounts of C18:3 *n*-6 and C20:4 *n*-6, for the omega-6 series, and C18:3 *n*-3, C20:3 *n*-3, and C22:6 *n*-3 for the omega-3 one in the two treatments.

Scientific literature [34] showed that an increase in yolk omega-3 FA is generally accompanied by a decrease in PUFA, especially arachidonic acid (C20:4 *n*-6). The DHA is a fundamental FA for human health since it is involved in brain and eye development, in regulating gut microbiota, preventing cardiovascular diseases, etc. [35]. In fact, the World Health Organization recommends a daily intake of 250 mg EPA + DHA [36]. Diversely to DHA, whose amount in the eggs of hens fed CS was 86 mg/100 g egg after 28 days, EPA was not detected in significant amounts. This was expected since it had been observed that EPA is immediately converted to docosapentaenoic acid (C22:5 *n*-3) and DHA in the liver before it is transported to the yolk [37].

Results of the present experiment indicated that the plateau for incorporating ALA into the egg was reached in 5 weeks, which exceeded 1 week that of EPA + DHA. This discrepancy could be attributed to the different metabolic expenditure required by ALA vs. EPA + DHA. While ALA is already present in the feedstuff, EPA + DHA are the result of a more complex enzymatic metabolic pathway which, after 4 weeks, probably reached the upper threshold limit. The time required to reach the plateau of omega-3 FA incorporation into the egg found confirmation in the existing literature [38,39], and, specifically, it fell within the 24–43-day period reported for different dietary omega-3 FA inclusion levels [40]. The time required to reach the plateau, as well as the absolute final amount, is known to be affected by the dietary omega-3 FA content [39], as well as by the above-discussed metabolic implications associated with the presence of antinutritional factors in the tested feedstuff and the consequent negative effects on nutrient absorption.

The shelf-life of CS eggs followed a similar evolution to the Control ones: pH increased during storage as a result of the breaking down of the ovomucin–lysozyme complex [41] and egg yolk was partly discolored, possibly due to pigment degradation and pH changes [42]. Interestingly, at Day 28 of shelf-life, CS eggs displayed a higher lipid peroxidation compared to Control eggs, but not at Day 0. This result shows that the lipid peroxidation was not simply ascribable to a higher deposition of oxidized lipids from the diet [31], but it confirms that, during storage, egg lipids can be further oxidized [43]. This result was partly unexpected since camelina is known to contain relevant amounts of phenolic compounds and flavonoids [44], which should have protected eggs against oxidative phenomena. The observed result could be ascribable to a combination of the following factors: (a) the processing step (pressing) to separate the oil and meal fractions from camelina seeds, which could have led to a slight oxidative deterioration of lipids, especially the omega-3 FA fraction, (b) a depletion of camelina phenolic compound and flavonoids during cake storage, (c) a higher susceptibility of CS eggs to lipid oxidation than Control ones due the higher content of omega-3 FA. Coherently with our results, Cherian et al. [13] observed an increase in peroxidation of egg lipids with increasing dietary incorporation of *Camelina sativa* meal (5, 10, 15%). In this regard, the addition of an extra amount of vitamin E or other antioxidants in the diet could be beneficial in protecting dietary lipids during storage and egg lipids from oxidative phenomena during retail display [45].

At the end of the laying season, the meat of laying hens also benefited from the CS diet, since the content of ALA and of the total omega-3 FA showed a 3-fold increase compared to the Control group. This was, however, insufficient to reach any nutritional claim due to the extremely low lipid content of the meat (average lipids for Control and CS hens: 1.79 g/100 g meat). This was very similar to what it was observed by Dalle Zotte et al. [46] on the breast meat of quails fed with 15% camelina cake, where the extremely low lipid content of the breast did not allow to appreciate a notable amount of omega-3 fatty acids, despite the proportional increase being relevant (the omega-3 FA % of camelina treatments was 7–8 times that of the quails belonging to the control group). While there are no studies testing the impact of dietary *Camelina sativa* on the FA profile of spent laying hens, different research has been conducted on broiler chickens [47,48,49]. It was generally observed an inclusion level-dependent improvement in the omega-3 FA content of meat of camelina-fed birds [48,49]. The greatest omega-3 PUFA deposition was achieved when feeding a diet incorporated with 24% camelina cake for 42 days, as meat reached the nutritional claim for omega-3 FA (349 and 674 mg/100 g breast and thigh meat omega-3 FA, respectively) [47].

## 5. Conclusions

The present research demonstrated that the incorporation of 5% *Camelina sativa* (CS) cake and 1% oil into the diet for laying hens allows to obtain eggs with the nutritional claim “rich in omega-3 fatty acids”, in accordance with the Regulation (EC) No. 1924/2006 of the European Parliament and Council of 20 December 2006 on nutrition and health claims made on foods. This result was achieved with a feeding period of 28 days, after which the deposition of EPA + DHA in the egg yolk reached a plateau. Concerning the other qualitative indicators considered in the present research (physical characteristics, proximate composition, and shelf-life), CS eggs were overall comparable to Control ones. Lastly, the meat of hens at the end of the laying season was enriched in omega-3 fatty acids too, but the low amount of lipids in the carcass did not allow for the obtainment of any nutritional claim. Further research could focus on assessing the economic and productive feasibility of a dietary implementation: providing a higher CS inclusion and enhancing the energy level to obtain a meat richer in beneficial lipids. The perspective would be to obtain a nutritional claim also for the hen’s meat without penalizing performance.

## Figures and Tables

**Table 1:** Chemical composition (g/kg as is) and gross energy content (MJ/kg) of the experimental diets.

Items	Experimental Diets	
	Control	CS
Dry matter	908	922
Crude protein	146	145
Crude fibre	37.2	53.7
Ether extract	31.8	41.9
Starch	260	240
Ash	48.3	98.3
Ca, mg/kg	31,447	36,075
P, mg/kg	6971	6215
Ca/P	4.51	5.80
Gross energy	16.6	16.0

**Table 2:** Fatty acids profile (% of total FAME) of the experimental diets for laying hens.

Items	Experimental Diets	
	Control	CS
C14:0	0.15	0.09
C15:0	0.06	0.12
C16:0	14.4	11.3
C17:0	0.10	0.07
C18:0	2.41	2.47
C20:0	0.41	0.91
ΣSFA	17.5	15.1
C14:1 *n*-9	0.06	0.10
C15:1 *n*-9	0.11	0.00
C16:1 *n*-9	0.24	0.16
C18:1 *n*-9	24.7	19.7
C18:1 *n*-7	0.99	0.94
C20:1 *n*-9	0.45	4.88
C22:1 *n*-9	0.00	1.09
ΣMUFA	26.1	22.0
C18:2 *n*-6	51.6	42.5
C18:3 *n*-6	0.00	0.27
C18:3 *n*-3	3.37	12.3
C20:2 *n*-6	0.06	0.67
C20:4 *n*-6	0.00	0.37
C20:5 *n*-3	0.23	0.33
C22:2 *n*-6	0.08	0.19
C22:6 *n*-3	0.24	0.27
ΣPUFA	56.0	61.9
Σ*n*-6	51.7	44.0
Σ*n*-3	3.85	12.9
*n*-6/*n*-3	13.4	3.40
Identified, %	99.6	99.0

**Table 3:** Proximate composition (g/100 g, as is) of the eggs at Day 1 and Day 28 of the feeding period.

Items	Experimental Groups		RSD ^1^	*p*-Value
	Control	CS		
*Day 1*				
N. eggs	12	12		
Water	75.9	76.0	0.59	0.7525
Protein	15.3	15.4	0.26	0.4632
Lipids	7.91	7.75	0.52	0.4857
Ash	0.86	0.85	0.02	0.5776
*Day 28*				
N. eggs	12	12		
Water	76.2	76.1	0.66	0.7308
Protein	15.0	15.0	0.32	0.7252
Lipids	8.01	8.07	0.59	0.8373
Ash	0.86	0.85	0.04	0.7980

^1^ RSD: residual standard deviation.

**Table 4:** FA content (mg/100 g egg) of the eggs at the beginning of the feeding period.

Items	Experimental Groups		RSD ^1^	*p*-Value
	Control	CS		
N. eggs	12	12		
C4:0	0.00	0.79	1.53	0.2585
C6:0	1.00	1.84	1.80	0.0524
C8:0	0.00	1.19	1.53	0.0948
C10:0	0.49	0.68	1.86	0.8150
C12:0	0.78	2.01	2.83	0.3371
C14:0	25.3	24.7	3.90	0.7398
C15:0	7.71	6.18	2.17	0.1247
C16:0	1899	1819	140	0.2112
C17:0	21.5	20.0	2.66	0.2306
C18:0	581	610	52.1	0.2283
C20:0	0.00	0.72	1.40	0.2585
ΣSFA	2535	2486	184	0.5537
C14:1	0.95	0.79	2.28	0.8718
C15:1	0.45	0.76	1.90	0.7179
C16:1	169	167	21.8	0.8212
C17:1	10.1	10.0	1.78	0.8927
C18:1 *n*-9	2999	2955	212	0.6422
C18:1 *n*-11	131	130	11.0	0.8328
C20:1 *n*-9	21.7	25.1	2.34	0.0700
ΣMUFA	3332	3288	235	0.6766
C18:2 *n*-6	1432	1360	170	0.3468
C18:3 *n*-6	12.2	11.3	1.48	0.1839
C20.2 *n*-6	17.0	18.9	1.97	0.0533
C20:3 *n*-6	9.95	10.5	1.05	0.0676
C20:4 *n*-6	89.5	95.2	12.1	0.2993
C22:2 *n*-6	1.70	2.92	3.28	0.4111
C18:3 *n*-3	74.1	72.8	12.6	0.8148
C20:5 *n*-3	0.45	0.00	1.19	0.4003
C22:6 *n*-3	43.6	47.0	6.48	0.2470
ΣPUFA	1681	1619	185	0.4578
Σ*n*-6	1563	1499	173	0.4155
Σ*n*-3	118	120	14.1	0.7950
EPA + DHA	44.0	47.0	6.72	0.3304

^1^ RSD: residual standard deviation.

**Table 5:** Effect of a dietary inclusion with *Camelina sativa* cake and oil for 28 days on the FA content (mg/100 g egg) of the eggs.

Items	Experimental Groups		RSD ^1^	*p*-Value
	Control	CS		
N. eggs	12	12		
C14:0	25.6	22.8	3.24	0.0564
C15:0	6.28	6.73	0.88	0.2360
C16:0	1949	1837	139	0.0670
C17:0	19.2	20.9	2.98	0.1907
C18:0	639	618	62.4	0.4456
C20:0	0.41	1.07	1.96	0.4312
ΣSFA	2639	2508	185	0.1031
C14:1	4.17	1.86	3.00	0.0797
C16:1	173	166	26.0	0.5257
C17:1	8.73	8.66	1.26	0.8896
C18:1 *n*-9	2905	2887	266	0.8727
C18:1 *n*-11	126	121	13.3	0.4112
C20:1 *n*-9	18.9	44.2	4.66	<0.0001
ΣMUFA	3235	3230	299	0.9653
C18:2 *n*-6	1558	1477	128	0.1453
C18:3 *n*-6	10.8	8.65	1.36	0.0009
C20:2 *n*-6	18.7	19.7	2.18	0.2922
C20:3 *n*-6	9.81	9.13	1.06	0.1396
C20:4 *n*-6	114	74.4	15.7	<0.0001
C22:2 *n*-6	0.79	0.00	1.34	0.1712
C18:3 *n*-3	54.9	227	27.4	<0.0001
C20:3 *n*-3	0.00	8.86	1.24	<0.0001
C22:6 *n*-3	54.8	85.5	14.1	0.0004
ΣPUFA	1822	1902	150	0.2142
Σ*n*-6	1713	1589	132	0.0364
Σ*n*-3	110	313	31.4	<0.0001
EPA + DHA	54.8	85.5	14.3	0.0004

^1^ RSD: residual standard deviation.

**Table 6:** Effect of a dietary inclusion with *Camelina sativa* cake and oil for 28 days on the physical characteristics of eggs.

Items	Experimental Groups		RSD ^1^	*p*-Value
	Control	CS		
N. eggs	70	70		
*Whole egg:*				
Weight, g	59.1	59.5	5.13	0.6709
Surface area, cm^2^	70.0	71.1	4.32	0.6910
Height, mm	58.1	57.4	2.07	0.0748
Diameter, mm	42.9	43.2	1.34	0.1815
Shape index, %	73.9	75.3	2.28	0.0012
Fracture toughness ^2^, N	39.6	45.2	9.87	0.0024
Edible portion, %	80.7	80.3	2.22	0.3903
*Yolk:*				
Weight, g	16.3	15.9	1.84	0.2304
% egg weight	27.6	26.9	2.73	0.1524
*Albumen:*				
Weight, g	31.5	31.8	4.45	0.6871
% egg weight	53.1	53.4	3.53	0.5687
Yolk to albumen ratio	0.52	0.51	0.08	0.3919
*Shell:*				
Weight, g	6.73	6.90	0.76	0.2479
% egg weight	11.4	11.6	0.81	0.1713
Thickness, mm	0.44	0.45	0.03	0.1968

^1^ RSD: residual standard deviation; ^2^ Equatorial.

**Table 7:** Effect of the dietary inclusion of *Camelina sativa* on the 28-day shelf-life of laying hen eggs.

Items	Experimental Groups		RSD ^1^	*p*-Diet	*p*-Diet × Day
	Control	CS			
N. eggs	12	12			
*pH*					
Day 0	8.77	8.78	0.22	0.9636	0.9508
Day 28	9.37	9.37	0.07	0.9508	
RSD	0.14	0.19			
*p*-Day	<0.0001	<0.0001			
*Yolk Color Fan* ^2^					
Day 0	11.1	11.4	0.73	0.2776	0.5140
Day 28	9.58	10.3	1.09	0.1458	
RSD	0.95	0.90			
*p*-Day	0.0008	0.0063			
*TBARs * ^3^ *, mg MDA/kg egg*					
Day 0	0.77	0.75	0.04	0.1797	0.0076
Day 28	0.78	0.83	0.04	0.0190	
RSD	0.04	0.05			
*p*-Day	0.6026	0.0006			

MDA: Malondialdehyde; ^1^ RSD: residual standard deviation; ^2^ dsm-firmenich YolkFan^TM^; ^3^ Thiobarbituric acid reactive substances.

**Table 8:** Proximate composition (g/100 g, as is) of the meat obtained from hen’s carcasses at the end of the laying season.

Items	Experimental Groups		RSD ^1^	*p*-Value
	Control	CS		
N. hens	6	6		
Water	74.7	74.7	0.86	0.8804
Protein	22.3	21.6	0.29	0.0018
Lipids	1.51	2.06	0.73	0.2162
Ash	1.07	1.06	0.01	0.3948

^1^ RSD: residual standard deviation.

**Table 9:** Effect of a dietary inclusion with *Camelina sativa* cake and oil on the FA content of the meat obtained from hen’s carcasses at the end of the laying season (mg/100 g meat).

Items	Experimental Groups		RSD ^1^	*p*-Value
	Control	CS		
N. hens	6	6		
C14:0	0.58	0.59	0.48	0.9678
C15:0	2.02	2.22	0.41	0.4074
C16:0	303	388	133	0.2950
C17:0	2.42	3.54	1.38	0.1913
C18.0	167	211	64.4	0.2633
C20:0	7.12	21.9	17.3	0.1701
ΣSFA	482	627	211	0.2617
C14:1 *n*-9	0.59	0.49	0.51	0.7461
C16:1 *n*-9	28.8	36.8	12.6	0.2965
C17:1 *n*-9	0.86	1.26	0.72	0.3700
C18:1 *n*-9	310	578	243	0.0848
C18:1 *n*-7	114	34.5	106	0.2172
C20:1 *n*-9	0.00	1.13	0.59	0.0078
ΣMUFA	455	683	225	0.1594
C18:2 *n*-6	384	523	207	0.2714
C18:3 *n*-3	17.4	56.0	35.2	0.0476
C18:3 *n*-6	1.56	2.27	0.96	0.2284
C20:2 *n*-6	4.92	3.66	2.14	0.3308
C20:3 *n*-6	0.40	1.49	0.88	0.0578
C20:4 *n*-6	86.2	91.2	29.2	0.7770
C20:5 *n*-3	4.07	6.14	3.03	0.2640
ΣPUFA	498	683	260	0.2462
Σ*n*-6	477	621	233	0.3076
Σ*n*-3	21.5	62.1	38.1	0.0446

^1^ RSD: residual standard deviation.

## Data Availability

The raw data supporting the conclusions of this article will be made available by the authors on request.

## References

[1] FAO-Food and Agriculture Organization of the United Nations (2022). FAOSTAT Crops and Livestock Products. https://www.fao.org/faostat/en/#data/QCL.

[2] Sanlier N., Üstün D. (2021). Egg consumption and health effects: A narrative review. J. Food Sci..

[3] Molnár S., Szőllősi L. (2020). Sustainability and quality aspects of different table egg production systems: A literature review. Sustainability.

[4] European Commission (2020). Agriculture and Rural Development. https://agriculture.ec.europa.eu/farming/animal-products/eggs_en.

[5] Rahmani D., Kallas Z., Pappa M., Gil J.M. (2019). Are consumers’ egg preferences influenced by animal-welfare conditions and environmental impacts?. Sustainability.

[6] Sinclair M., Lee N.Y., Hötzel M.J., de Luna M.C.T., Sharma A., Idris M., Islam M.A., Iyasere O.S., Navarro G., Ahmed A.A. (2022). Consumer attitudes towards egg production systems and hen welfare across the world. Front. Anim. Sci..

[7] Palmieri N., Stefanoni W., Latterini F., Pari L. (2022). Factors influencing Italian consumers’ willingness to pay for eggs enriched with omega-3-fatty acids. Foods.

[8] Zanetti F., Alberghini B., Marjanović Jeromela A., Grahovac N., Rajković D., Kiprovski B., Monti A. (2021). Camelina, an ancient oilseed crop actively contributing to the rural renaissance in Europe. A review. Agron. Sustain. Dev..

[9] Juodka R., Nainienė R., Juškienė V., Juška R., Leikus R., Kadžienė G., Stankevičienė D. (2022). Camelina (*Camelina sativa* (L.) Crantz) as feedstuffs in meat type poultry diet: A source of protein and n-3 fatty acids. Animals.

[10] Singh Y., Cullere M., Tumová E., Dalle Zotte A. (2023). *Camelina sativa* as a sustainable and feasible feedstuff for broiler poultry species: A review. Czech J. Anim. Sci..

[11] Hajiazizi F., Sadeghi A., Ibrahim S. (2024). *Camelina sativa* (L. Crantz) products; an alternative feed ingredient for poultry diets with its nutritional and physiological consequences. Trop. Anim. Health Prod..

[12] Singh Y., Cullere M., Dalle Zotte A. (2023). *Camelina sativa* as a sustainable and feasible feedstuff for laying poultry: A review. Biotechnol. Anim. Husb..

[13] Cherian G., Campbell A., Parker T. (2009). Egg quality and lipid composition of eggs from hens fed *Camelina sativa*. J. Appl. Poult. Res..

[14] Kakani R., Fowler J., Haq A.U., Murphy E.J., Rosenberger T.A., Berhow M., Bailey C.A. (2012). Camelina meal increases egg n-3 fatty acid content without altering quality or production in laying hens. Lipids.

[15] Lolli S., Grilli G., Ferrari L., Battelli G., Pozzo S., Galasso I., Russo R., Brasca M., Reggiani R., Ferrante V. (2020). Effect of different percentage of *Camelina sativa* cake in laying hens diet: Performance, welfare, and eggshell quality. Animals.

[16] Orczewska-Dudek S., Pietras M., Puchała M., Nowak J. (2020). *Camelina sativa* oil and camelina cake as sources of polyunsaturated fatty acids in the diets of laying hens: Effect on hen performance, fatty acid profile of yolk lipids, and egg sensory quality. Ann. Anim. Sci..

[17] Razmaitė V., Šiukščius A., Leikus R. (2022). Effects of dietary rapeseed (*Brassica napus*), hemp (*Cannabis sativa*) and camelina (*Camelina sativa*) seed cakes supplementation on yolk and albumen colour and nutritional value of yolk lipids in Estonian quail eggs. Animals.

[18] Jin S.K., Kim I.S., Jung H.J., Kim D.H., Choi Y.J., Hur S.J. (2007). The development of sausage including meat from spent laying hen surimi. Poult. Sci..

[19] Kumar A., Mendiratta S.K., Sen A.R., Kandeepan G., Talukder S., Sharma H., Soni A., Irshad A., Kumar S. (2015). Preparation and storage stability of meat spread developed from spent hens. Vet. World.

[20] Fan H., Wu J. (2022). Conventional use and sustainable valorization of spent egg-laying hens as functional foods and biomaterials: A review. Bioresour. Bioprocess..

[21] AOAC (Association of Official Analytical Chemists) (2019). Official Methods of Analysis.

[22] European Commission Commission Directive 98/64/EC of 3 September 1998 Establishing Community Methods of Analysis for the Determination of Amino Acids, Crude Oils and Fats, and Olaquindox in Feeding Stuffs and Amending Directive 71/393/EEC. https://publications.europa.eu/en/publication-detail/-/publication/856db9b7-6f1d-4d6e-a778-c766c9fa1776.

[23] Dalle Zotte A., Singh Y., Palumbo B., Contiero B., Cullere M. (2024). Live yellow mealworm (*Tenebrio molitor*) larvae: A promising nutritional enrichment for laying quails. Poult. Sci..

[24] Dalle Zotte A., Cullere M., Pellattiero E., Sartori A., Marangon A., Bondesan V. (2021). Is the farming method (cage, barn, organic) a relevant factor for marketed egg quality traits?. Livest. Sci..

[25] Botsoglou N.A., Fletouris D.J., Papageorgiou G.E., Vassilopoulos V.N., Mantis A.J., Trakatellis A.G. (1994). Rapid, sensitive, and specific thiobarbituric acid method for measuring lipid peroxidation in animal tissue, food, and feedstuff samples. J. Agric. Food Chem..

[26] SAS—Statistical Analysis Software for Windows (2008). Statistics Version 9.1.3.

[27] Delver J.J., Smith Z.K. (2024). Opportunities for Camelina Meal as a Livestock Feed Ingredient. Agriculture.

[28] Altuntaş E., Şekeroğlu A. (2008). Effect of egg shape index on mechanical properties of chicken eggs. J. Food Eng..

[29] Severa L., Nedomová Š., Buchar J., Cupera J. (2013). Novel approaches in mathematical description of hen egg geometry. Int. J. Food Prop..

[30] Jiang S., Cui L., Shi C., Ke X., Luo J., Hou J. (2013). Effects of dietary energy and calcium levels on performance, egg shell quality and bone metabolism in hens. Vet. J..

[31] Fraeye I., Bruneel C., Lemahieu C., Buyse J., Muylaert K., Foubert I. (2012). Dietary enrichment of eggs with omega-3 fatty acids: A review. Food Res. Int..

[32] Beheshti Moghadam M.H., Cherian G. (2017). Use of flaxseed in poultry feeds to meet the human need for n-3 fatty acids. World’s Poult. Sci. J..

[33] Guo W.L., Fu S.S., Li X.Y., Wang C.C., Xue C.H., Wang Y.M., Zhang T.T. (2025). Fucoxanthin promotes the conversion efficiency of alpha-linolenic acid in feeding to docosahexaenoic acid in quail egg yolk. Food Chem..

[34] Hayat Z., Cherian G., Pasha T.N., Khattak F.M., Jabbar M.A. (2009). Effect of feeding flax and two types of antioxidants on egg production, egg quality, and lipid composition of eggs. J. Appl. Poult. Res..

[35] Li J., Pora B.L., Dong K., Hasjim J. (2021). Health benefits of docosahexaenoic acid and its bioavailability: A review. Food Sci. Nutr..

[36] Colombo S.M., Rodgers T.F., Diamond M.L., Bazinet R.P., Arts M.T. (2020). Projected declines in global DHA availability for human consumption as a result of global warming. Ambio.

[37] Neijat M., Eck P., House J.D. (2017). Impact of dietary precursor ALA versus preformed DHA on fatty acid profiles of eggs, liver and adipose tissue and expression of genes associated with hepatic lipid metabolism in laying hens. Prostaglandins Leukot. Essent. Fat. Acids.

[38] Lemahieu C., Bruneel C., Termote-Verhalle R., Muylaert K., Foubert I., Buyse J. (2015). Dynamics of omega-3 long chain polyunsaturated fatty acid incorporation in egg yolk by autotrophic microalgal supplementation. Eur. J. Lipid Sci. Technol..

[39] Maina A.N., Lewis E., Kiarie E.G. (2023). Egg production, egg quality, and fatty acids profiles in eggs and tissues in Lohmann LSL lite hens fed algal oils rich in docosahexaenoic acid (DHA). Poult. Sci..

[40] Neijat M., Ojekudo O., House J.D. (2016). Effect of flaxseed oil and microalgae DHA on the production performance, fatty acids and total lipids of egg yolk and plasma in laying hens. Prostaglandins Leukot. Essent. Fat. Acids.

[41] Dalle Zotte A., Singh Y., Michiels J., Cullere M. (2019). Black soldier fly (*Hermetia illucens*) as dietary source for laying quails: Live performance, and egg physico-chemical quality, sensory profile and storage stability. Animals.

[42] Kljak K., Carović-Stanko K., Kos I., Janječić Z., Kiš G., Duvnjak M., Safner T., Bedeković D. (2021). Plant carotenoids as pigment sources in laying hen diets: Effect on yolk color, carotenoid content, oxidative stability and sensory properties of eggs. Foods.

[43] Mohiti-Asli M., Shariatmadari F., Lotfollahian H., Mazuji M.T. (2008). Effects of supplementing layer hen diets with selenium and vitamin E on egg quality, lipid oxidation and fatty acid composition during storage. Can. J. Anim. Sci..

[44] Tavarini S., De Leo M., Matteo R., Lazzeri L., Braca A., Angelini L.G. (2021). Flaxseed and camelina meals as potential sources of health-beneficial compounds. Plants.

[45] Migliorini M.J., Boiago M.M., Stefani L.M., Zampar A., Roza L.F., Barreta M., Arno A., Robazza W.S., Giuriatti J., Galvão A.C. (2019). Oregano essential oil in the diet of laying hens in winter reduces lipid peroxidation in yolks and increases shelf life in eggs. J. Therm. Biol..

[46] Dalle Zotte A., Singh Y., Pellattiero E., Palumbo B., Cullere M. (2024). Different lines of camelina (*Camelina sativa* (L.) Crantz) in broiler quails’ diets: Effects on meat physicochemical traits and sensory profile. Ital. J. Anim. Sci..

[47] Nain S., Oryschak M.A., Betti M., Beltranena E. (2015). *Camelina sativa* cake for broilers: Effects of increasing dietary inclusion from 0 to 24% on tissue fatty acid proportions at 14, 28, and 42 d of age. Poult. Sci..

[48] Ciurescu G., Ropota M., Toncea I., Habeanu M. (2016). Camelia (*Camelina sativa* L. Crantz Variety) oil and seeds as n-3 fatty acids rich products in broiler diets and its effects on performance, meat fatty acid composition, immune tissue weights, and plasma metabolic profile. J. Agr. Sci. Tech..

[49] Orczewska-Dudek S., Pietras M. (2019). The effect of dietary *Camelina sativa* oil or cake in the diets of broiler chickens on growth performance, fatty acid profile, and sensory quality of meat. Animals.

